# Guidelines and tumor boards are required for solid organ recipients with *de novo* carcinoma

**DOI:** 10.18632/oncotarget.26860

**Published:** 2019-04-19

**Authors:** Benoit Rousseau, Aude Guillemin, Christophe Tournigand

**Affiliations:** Benoit Rousseau: Department of Medicine, Solid Tumor Division, Mortimer B. Zuckerman Research Center, Memorial Sloan Kettering Cancer Center, New York, NY, USA; Medical Oncology, Henri Mondor Hospital, Assistance Publique-Hôpitaux de Paris, Créteil, France; University of Paris-Est, Faculty of Medicine, Créteil, France

**Keywords:** cancer, transplantation, prognosis, mTOR inhibitors, guidelines

In 2015, 126,670 solid organ transplantations were carried out worldwide [1] and the number of patients living with a functional transplant grows up [2]. Risk of cancer after solid organ transplantation is increased by two to three fold compared to general population [3]. The cumulative incidence of cancer after transplantation reaches 9% at 10 years [4]. This higher risk is related to patient intrinsic factors, such as cause for transplantation, way of life, comorbidities, along with intense and/or prolonged immunosuppression modifying the balance between immune tolerance and anti-tumoral immunity [5]. Transplanted patients with de novo carcinoma are therefore a difficult to treat population because oncologic management would have to consider the comorbidities and risks of graft rejection and/or impairment, limiting therapeutic options. Specifically, in this population, next generation immunotherapies such as immune checkpoints inhibitors may result in life-threatening acute graft rejection [6].

In a large real-life cohort of 4637 kidney and liver allograft recipients, we identified 205 cases of de novo cancer in 180 patients and investigated the impact of optimal oncologic management according to oncologic guidelines and immunosuppressive therapy modifications on survival and toxicities [7]. We showed that oncologic treatments are feasible in this population without increased risk of graft rejection, and that transplantation/immunosuppression hindered optimal oncologic management in only 11% instances. In 46% of cases, immunosuppressive therapy was modified after cancer diagnosis with 24% dose reduction and 22% mTOR inhibitor introduction. Optimal oncologic treatment was performed in 80% and 37% of patients with localized and advanced cancer respectively. In multivariate analysis, mTOR introduction after de novo cancer was associated with a reduction of 74% of death risk (HR = 0.26; 95% CI 0.01-0.58; *P* = 0.001), and optimal oncologic treatment with a 55%-reduction of death risk (HR = 0.45; 95% CI: 0.01-0.99, *P* = 0.047). This effect was observed whatever the stage of the disease. Notably, dose reduction of immunosuppressant drugs was not associated with improved prognosis suggesting a mTOR inhibitors class effect due to their direct antitumoral effect and/or indirect immunomodulation properties.

These results highlight the need to propose the best possible oncologic strategies to solid organ transplanted patient with *de novo* carcinoma as they display a high benefit from recommended treatments. Nevertheless, medical and radiation oncologists or surgeons would have to consider the risk of antitumoral treatment-related toxicities on the graft and a joint assessment with transplant specialists should be performed. The question of modification of immunosuppressant regimen should also be addressed as our study, along with others [8, 9], underlies that immunosuppressive therapy discontinuation, dose reduction, or switch to an mTOR inhibitor may help to restore anti-tumoral immune responses and improve survival. Given the numerous clinical settings, such a tumor type and stage or intensity of immunosuppression which differs from one graft to another, we would like to propose that dedicated guidelines for solid organ recipients with *de novo* carcinomas are urgently needed both for oncologic management and immunosuppressant-sparing strategies. As shown in our study for transplanted patients with advanced cancers, only 37.5% of patients received optimal treatment, and 27.0% of patients had exclusive best supportive care as oncologists may be reluctant and afraid to offer systemic therapies to these comorbid patients at risk of toxicities. Dedicated guidelines may help to reduce this effect, providing better access to optimal oncologic care. Moreover, to our knowledge, there is no consensus on immunosuppressive therapy modification after *de novo* cancer in transplanted patients. Our study showed that less than half of transplanted patients with de novo cancer had a decrease in the intensity of immunosuppression or a switch to mTOR inhibitors. A systematic review of the literature performed by experts and aiming to provide guidelines on immunosuppressive strategies in this clinical setting would be of added value.

Another way to improve oncologic care in this specific population, would be to create specific tumor board for transplanted patients with the participation of cancer and transplant specialists. It has been shown that specialized tumor boards for rare tumors improve the quality of care and survival as they concentrate the cases, implicate highly trained specialists in the field and help to comply with the clinical practice guidelines [10]. It would also be a convenient way to develop national and international cohorts, a preliminary condition for upcoming clinical trials aiming to investigate innovative strategies in solid organ recipients with *de novo* carcinomas.
